# Digital PCR in Myeloid Malignancies: Ready to Replace Quantitative PCR?

**DOI:** 10.3390/ijms20092249

**Published:** 2019-05-07

**Authors:** Daniela Cilloni, Jessica Petiti, Valentina Rosso, Giacomo Andreani, Matteo Dragani, Carmen Fava, Giuseppe Saglio

**Affiliations:** Department of Clinical and Biological Sciences, University of Turin, 10043 Turin, Italy; jessica.petiti@unito.it (J.P.); valentina.rosso@unito.it (V.R.); giacomo.andreani@unito.it (G.A.); matteo.dragani@gmail.com (M.D.); carmen.fava@unito.it (C.F.); giuseppe.saglio@unito.it (G.S.)

**Keywords:** digital PCR, acute myeloid leukemia, chronic myeloproliferative disorders, minimal residual disease

## Abstract

New techniques are on the horizon for the detection of small leukemic clones in both, acute leukemias and myeloproliferative disorders. A promising approach is based on digital polymerase chain reaction (PCR). Digital PCR (dPCR) is a breakthrough technology designed to provide absolute nucleic acid quantification. It is particularly useful to detect a low amount of target and therefore it represents an alternative method for detecting measurable residual disease (MRD). The main advantages are the high precision, the very reliable quantification, the absolute quantification without the need for a standard curve, and the excellent reproducibility. Nowadays the main disadvantages of this strategy are the costs that are still higher than standard qPCR, the lack of standardized methods, and the limited number of laboratories that are equipped with instruments for dPCR. Several studies describing the possibility and advantages of using digital PCR for the detection of specific leukemic transcripts or mutations have already been published. In this review we summarize the available data on the use of dPCR in acute myeloid leukemia and myeloproliferative disorders.

## 1. Introduction

Polymerase chain reaction (PCR) is a revolutionary method for DNA amplification set up in 1985 by Kary Mullis, that allows the quantification of nucleic acids by amplification with the enzyme DNA polymerase [1].

The conventional assay is based on the principle that amplification of the nucleic acid is exponential thus it can be quantified by comparing the number of amplification cycles and the amount of PCR end-products to those of a reference sample [2,3,4].

In a conventional PCR, a solution of template DNA, DNA polymerase, deoxyribonucleotide triphosphates (dNTPs), primers, and buffer solution are subjected to a series of thermal cycles to amplify millions of copies of a template DNA [2,3,4].

In the current modified version of the PCR, the first step of a thermal cycle begins with a process called initialization or hot start to activate the polymerase at a temperature ranging from 94 °C to 96 °C. This step is followed by a denaturation step at high temperature between 93 °C and 98 °C. This step breaks the bonds in double stranded DNA (dsDNA) thus generating two single-stranded DNA (ssDNA) molecules. After denaturation, a phase called annealing starts at lower temperature. Primers are annealed to complementary sequences of the single-stranded DNA molecule. Subsequently the PCR mixture is heated to allow incomplete DNA sequence to be extended by the DNA polymerase in the presence of free dNTPs thus generating a new double-stranded DNA.

Despite the high level of sensitivity of this method, the evaluation of the amount of transcript can be hampered by many factors that can lead to inaccuracies. Among these, the low initial concentration of target molecules may not be amplified to detectable levels. In addition, the PCR amplification product can reach a plateau after a certain number of cycles. Finally, PCR amplification efficiency can differ in different samples. All these limits can impact on the accuracy of the quantification of the nucleic acid.

Real Time quantitative PCR (qPCR) represents the evolution of standard PCR. With this technique the products are continuously monitored throughout the reaction cycles using fluorescent dyes [5].

The initial amount of DNA template can be established by comparing the fluorescence output curve of the qPCR with a standard curve generated with different known initial numbers of DNA copies. The cycle threshold (Ct) is defined as the number of cycles required for the fluorescent signal to cross the threshold and to become detectable. Ct levels are inversely proportional to the amount of target nucleic acid in the sample.

The qPCR technique is more accurate than standard PCR and finds application in molecular diagnosis of many hematological malignancies and in the detection of a low amount of residual disease [6].

Nowadays, due to the high level of sensitivity, the qPCR technique, together with multiparameter flow cytometry (MFC) is considered the gold standard for the detection of malignant cells in different types of hematological malignancies [6].

## 2. Digital PCR

Digital PCR (dPCR) represents an innovative evolution of qPCR with many practical advantages over qPCR. Although initially described in 1999 [7] by Vogelstein and Kinzler, dPCR has become only recently a reliable and applicable tool. The first report [7] described the successful detection of *Ras* mutations with a high level of sensitivity in a cohort of patients with colorectal cancer [7]. With this method the exponential signal of PCR is converted into a linear digital signal which is most suitable for identification of genetic mutation.

From the first report in 1999, dPCR has been successfully applied to cancer genome studies. In the last few years the interest for this method in the hematological setting has progressively increased as testified by the number of papers in literature reporting the usefulness of this method for the quantification of specific leukemic aberrations. The main applications include evaluations of gene expression (e.g., miRNAs), pathogen quantification, rare allele detection, germline and somatic copy number variation estimation, viral load analysis, and microbial quantification [8].

In dPCR, the polymerase chain reaction mixture along with the necessary fluorophore is compartmentalized into several smaller units, each unit undergoes the same thermal cycles as in the case of a conventional PCR. Usually, dPCR employs the same primer sets, fluorescent labels, and enzymatic reagents as for traditional RT-PCR, unless recommended differently by the manufacturers. The key element of dPCR is the partition of the sample into thousands of individual PCR reactions in essence generating a limiting dilution [9]. As for RT-PCR, dPCR offers a highly precise and sensitive approach with the main advantage over RT-PCR of avoiding the need for a reference or standard curve. Despite this, it is necessary to admit that the same advantage can be obtained by performing a duplex-PCR with the inclusion of a reference gene.

**Figure 1:**
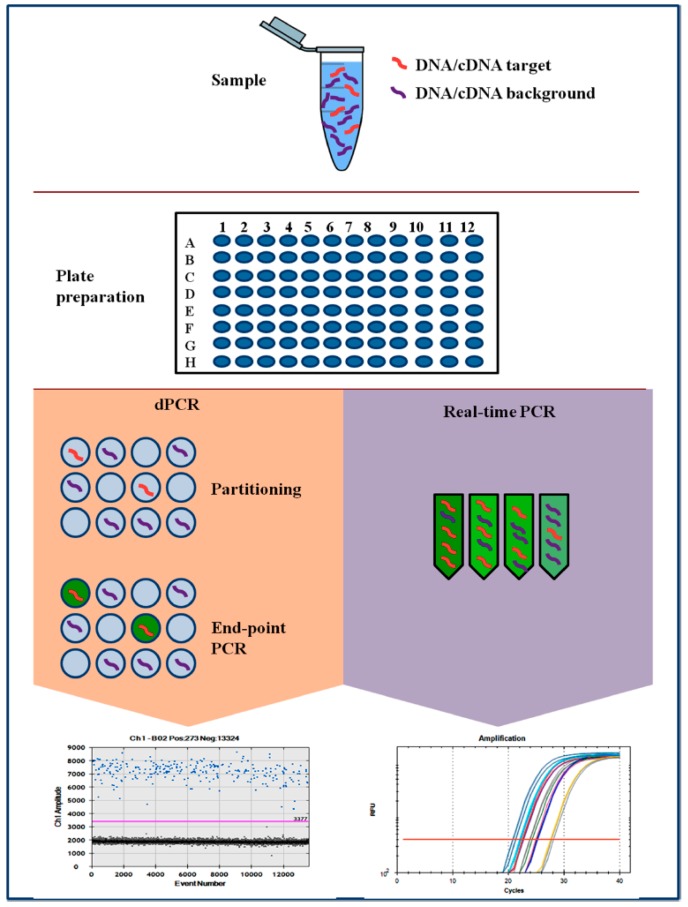
Comparison of PCR-based techniques. RT-PCR and dPCR using the same amplification reagents and fluorescent labeling system. In dPCR, the sample is first partitioned such that each partition contains either a few or no DNA sequences. Fluorescence is measured at the end of the PCR. In qPCR, the amount of amplified DNA is measured at each cycle during the PCR reaction.

Digital PCR method is based on three main points: the compartmentalization of the target, PCR on each single molecule and Poisson statistics (Figure 1). Following partition and amplification, the negative fraction is used to generate an absolute count of the number of target molecules in the sample, all without reference to standards or controls [10]. Nowadays, different commercialized digital PCR platforms are available. The first is based on Chip in a tube design (BioRad-QX200 digital PCR System, Bio-Rad system, Hercules, CA, USA). The second tool is based on micro-well chip (Life Technologies-QuantStudio3D^®^ Digital PCR, Life Technologies, Carlsbad, CA, USA). An additional platform is based on the microfluidic-chamber (Stilla Technologies-Naica Crystal dPCR, Villejuif, France and Fluidigm-BioMark^®^ HD, Fluidigm Corporation, San Francisco, CA, USA). Micro-well chip-based and microfluidic-chamber-based (cdPCR) technology can contain up to a few thousand individual reactions for each sample. Droplet dPCR (ddPCR) is a method based on emulsion PCR. The sample is fractionated into 20,000 droplets and the amplification of the template molecules occurs in each droplet [11]. The high partition of ddPCR, makes this method very sensitive and potentially useful for both, research and diagnostic purposes. The main advantages of dPCR compared to RT-PCR are the high precision, the very reliable quantification, the absolute quantification without the need for a standard curve, and excellent reproducibility [12].

Finally, recent papers have reported the higher tolerance of dPCR as compared to RQ-PCR to different types of inhibitors that can damage DNA or make DNA less accessible, like salts including KCl and NaCl, ionic detergents such as sodium deocycholate, sarkosyl, and SDS, ethanol, isopropanol, and phenol among others. This is mainly due to the compartmentalization of target sequences in smaller volumes [8,13].

An increasing number of manuscripts is published every month on the use of dPCR in hematological diseases. In this review we summarize the current knowledge on dPCR in myeloid neoplasms.

## 3. dPCR in Chronic Myeloproliferative Disorders: The Example of Chronic Myeloid Leukemia (CML)

In chronic myeloid leukemia (CML), the presence of a specific marker, the Philadelphia chromosome, together with the corresponding molecular marker (*BCR-ABL* fusion transcripts) provides a unique opportunity for the monitoring of the disease, at diagnosis and during therapy [14]. Lots of data clearly show that in CML both, cytogenetic and molecular responses, are strictly correlated to the final outcome and survival of the patients [15,16]. The use of adequate and standardized methods allows the degree of responses and the depth of disease reduction at specific time points during treatment to be established [17,18,19]. This has become fundamental for a correct clinical management of CML patients. The efforts to standardize the molecular monitoring and the criteria of response led to the definition of “optimal response” that may represent not only the highest probability of survival but also the possibility to discontinue the tyrosine-kinase inhibitor (TKI) treatment and therefore to live in a treatment-free remission (TFR) status [20,21]. Therefore, at least for some patients, deep degrees of molecular response, as MR4 and MR4.5, whose precise definition has been recently introduced and which are prerequisites to try discontinuation, are becoming the target to be achieved even in common clinical practice [15].

Currently, both European Leukemia Net (ELN) [15] and National Comprehensive Cancer Network (NCCN) [21] recommend the use of qPCR to monitor MRD or to establish the depth of molecular response.

With the aim of improving PCR sensitivity to increase the predictive value of molecular response, several studies compared the use of dPCR and qPCR. In the study published by Goh and colleagues, [22] forty-three CML patients were screened by conventional qPCR, by replicate qPCR (rRQ-PCR) and by nanofluidic digital PCR. Interestingly they demonstrated that rRQ-PCR, as well as dPCR with pre-amplification, has a 2–3 log of improvement compared to conventional RQ-PCR. Twenty-four of 32 PCR negative samples as assayed by conventional qPCR showed detectable *BCR–ABL* in rRQ-PCR and/or dPCR. Furthermore they were able to demonstrate that, using dPCR with a pre-amplification step, the progressive reduction of MRD level could be precisely monitored even when undetectable by conventional qPCR.

In summary, in this study, both rRQ-PCR and ddPCR succeed in the detection of *BCR–ABL* transcripts not detectable in conventional RQ-PCR.

These data showed the potential feasibility of highly sensitive PCR approaches for molecular monitoring and suggested the clinical relevance for future CML management by allowing further characterization of patients who achieve PCR negativity by qPCR and who are candidates for therapy discontinuation.

In 2018 Wang and colleagues [23] analyzed the peripheral blood samples from 61 CML patients who presented an undetectable level of *BCR-ABL* by qPCR after tyrosine kinase inhibitor (TKI) treatment in three successive analyses performed once every 3 months. In parallel, they measured the fusion transcript by droplet d-PCR and they found that 18% of the patients tested positive by this analysis. Importantly, during follow up they documented that the few cases who lost MR4 presented a markedly increased transcript amount, detected by dd-PCR, a few months before the loss of response. This increase was not detectable by conventional qPCR. These data led to the initial conclusion that dd-PCR is more sensitive in measuring deep molecular responses.

Different from what is reported above, the study by Alikian and colleagues [24], by analyzing a cohort of 70 CML patients, demonstrated that dPCR has a comparable performance to qPCR for the quantification of *BCR-ABL1*. Their conclusion is that qPCR already has a high level of sensitivity, close to the single molecule level.

## 4. Digital PCR in Philadelphia Negative Chronic Myeloproliferative Neoplasms (Ph-MPN)

Philadelphia-negative chronic myeloproliferative neoplasms (MPNs) are clonal disorders that present *JAK2^V617F^* mutation in a percentage ranging from 50% to 95% of cases [25]. Calreticulin (*CALR*) gene mutations have recently been discovered in about 20%–35% of patients affected by essential thrombocythemia (ET) and primary myelofibrosis (PMF) [26]. An additional percentage of ET patients presents with *MPL* mutation [27].

*JAK2*, *CALR*, and *MPL* “driver” mutations can be targeted for diagnosis and sometimes to monitor the disease response (i.e., after allogeneic stem cell transplantation) although the role of these markers in the evaluation of clinical response to currently available therapies is still questionable.

Due to the lack of effective therapies, able to induce molecular remission in MPN patients, the development of techniques for the detection of MRD has not been heavily pushed. Recently, a new scenario is on the horizon with new targeted therapies available or under development [27].

Furthermore, for myelofibrosis, the allogeneic hematopoietic stem cell transplantation (HSCT) might be curative [28]. In all these settings a precise evaluation of the kinetics of the disease might provide relevant information for treatment choices [29].

The clinical relevance of determining *JAK2*, *CALR*, and *MPL* based MRD has been demonstrated with several therapeutically approaches including interferon alpha, JAK1/2 inhibitors, and allogeneic stem cell transplantation [30].

Recently few studies have been published comparing qPCR and dPCR for the measurement of *JAK2^V617F^* [31,32,33].

In the study by Fontanelli [31] and colleagues, 99 patients affected by MPN with *JAK2^V617F^* were evaluated in parallel by means of qPCR and droplet dPCR. This latter showed a higher sensitivity than qPCR in detecting the mutation. They also confirmed an increased mutant allele burden from ET to PV and PMF.

Link-Lenczowska and colleagues were able to substantially confirm this finding [32]. They analyzed 63 MPN patients, six of them treated with ruxolitinib, by qPCR and dPCR. Basically, they found high concordance between the two methods, both demonstrated to be highly sensitive and both were capable of detecting the *JAK2^V617F^* mutation at diagnosis of MPN with a limit of detection of 0.12% for qPCR and 0.01% for ddPCR. The study suggests an advantage of ddPCR in monitoring MRD when the allele burden is below the threshold of detection of qPCR.

Finally, the clinical utility of dPCR for *JAK2^V617F^* mutation was investigated by Waterhouse and colleagues [33] in 59 patients with MPN, many of them after hematopoietic stem cell transplantation. The limit of detection was 0.01% for both qPCR and ddPCR. The *JAK2^V617F^* mutation was detected in 43 out of 59 patients by both PCR platforms. However, in 14% of the samples, *JAK2^V617F^* mutation was detected only with ddPCR. Interestingly, all these patients were analyzed shortly after allogeneic HSCT. Although available data on dPCR for *JAK2* detection after HSCT are still immature, this study suggests an intriguing role of dPCR in the context of myeloproliferative disorders. Similar to *JAK2^V617F^* mutation, the monitoring of *CALR* mutation might improve the therapeutic strategies. Mansier and colleagues [34] developed a digital PCR technique that allowed detection of types 1 and 2 *CALR* allelic burdens. They found that compared with the commonly used fluorescent PCR analysis, digital PCR is more precise, reproducible, and accurate. In their hands this method reached a very high sensitivity being able to detect at least 0.025% *CALR* mutants. They applied this method to patients with primary myelofibrosis who underwent hematopoietic stem cell transplant and they were able to predict relapse according to the reappearance of *CALR* mutations after HSCT. After the achievement of dPCR negativity, the reappearance of a low level of mutation, although in a single patient, preceded the hematologic relapse. This study suggests the possibility of using dPCR for CALR mutation measurement for MRD detection.

## 5. Minimal Residual Disease in Acute Myeloid Leukemias

Currently, the post remission treatment of patients affected by acute myeloid leukemias is based on the genetic profile of leukemic cells at diagnosis and on the level of minimal residual disease after induction and consolidation chemotherapy mainly detected by multiparameter flow cytometry and by q-PCR for the assessment of fusion transcripts levels (i.e., *CBFB-MYH11* and *RUNX1-RUNX1T1* and *PML-RARα*) of mutations, mainly *NPM1* [35,36]. The issue of MRD monitoring has been recently addressed by the European Leukemia Net (ELN) [6] in an attempt to standardize the methodology and to provide suggestion on when and how to monitor MRD.

A new promising approach for the detection of MRD is based on dPCR. We report below the current knowledge on the use of dPCR for detection of leukemic cells.

## 6. Digital PCR for Acute Myeloid Leukemia Monitoring: The Case of Acute Promyelocytic Leukemia

Acute promyelocytic leukemia (APL) is a hematological malignancy commonly associated with the chromosomal translocation t(15;17)(q24;q21), which involves the promyelocytic leukemia (*PML*) and the retinoic acid receptor-*α* (*RARα*) genes, resulting in the oncogenic fusion transcript *PML-RARα* [37].

In acute promyelocytic leukemia, the achievement of PCR negativity for *PML-RARα* at the end of consolidation treatment is the most informative in terms of prediction of outcome and it is associated with a low risk of relapse and a high probability of long-term survival [38,39]. The achievement of PCR negativity retains its clinical significance independently of the therapeutic strategies, all trans-retinoic acid (ATRA) associated with chemotherapy or ATRA and arsenic trioxide [38,39].

Recently Brunetti and colleagues [40] investigated the role of droplet dPCR for *PML-RARα* to monitor MRD in a cohort of 21 patients affected by APL. Droplet dPCR exhibited a sensitivity and specificity of 95% and 91% respectively for bcr1 and bcr3 transcripts and showed a good concordance with qPCR. The authors suggested that one of the main advantages of ddPCR-based monitoring of MRD is represented by the absolute quantification. They also believe that ddPCR could potentially provide crucial information for the management of patients whose MRD fluctuates under the level of detection of qPCR.

Similar results have been reported by Yuan and colleagues [41] in 28 APL patients. They confirmed the good concordance between ddPCR and qPCR in the detection of *PML-RARα* in clinical samples, but showed advantages of dPCR over qPCR in terms of precision, limit of detection, and other basic performance parameters.

In conclusion, based on the available data, at present dPCR could represent a complementary approach to monitor MRD in APL, particularly for those patients at high risk of relapse.

## 7. Acute Myeloid Leukemia with *IDH1/IDH2* Mutations

Since the evidence that *IDH1/IDH2* genes can be mutated in about 10%–20% of AML, many groups are investigating the possibility of using *IDH1/2* as a marker for MRD detection.

Until now, few data have been generated on the clearance of *IDH1/IDH2* after chemotherapy and during remission and contrasting data are reported in the literature [42,43]. Few studies reported the stability and suitability of *IDH* as a marker of MRD [42,44]. Recently Petrova and colleagues [45] published the evaluation of MRD in 90 patients, 22% of them with *IDH1/IDH2* mutations. They based the assessment on NGS and digital droplet PCR. Many patients presented additional mutations such as *NPM1* or *MLL-PDT*. They concluded that the persistence of *IDH1/2* correlates with the treatment response, although being less sensitive than *NPM1* in predicting relapse.

## 8. Monitoring *C-KIT* Exon 17 Mutations by Droplet Digital PCR in Patients with Core-Binding Factor AML

The cytogenetic aberrations involving core binding factor (CBF) include t(8;21)(q22;q22) and inv(16)(p13.1q22)/t(16;16) (p13.1;q22) [43]. CBF-AML are considered leukemias with a highly favorable prognosis although the outcome is highly impacted by the presence of additional genetic mutations, as exemplified by the mutation of *FLT3*, *C-KIT*, or *NRAS* gene [46,47,48,49]. In particular the mutations of *C-KIT* gene that result in the constitutive activation of tyrosine kinase activity have a significant impact on the prognosis of CBF acute myeloid leukemias. In particular, *C-KIT* mutations are associated with a higher incidence of relapse, so that CBF AML with *C-KIT* mutations are classified into the intermediate risk group by the NCCN Guideline [46,47,48,49]. These mutations are most frequently located in exon 17 (54%) encoding the kinase-activation loop, or in exon 8 (28%) affecting the extracellular portion of *C-KIT* receptor [46]. *C-KIT* mutations have been described initially as a single point mutation but the detection of double mutations has recently been described in AML patients [48].

Although we still lack solid data generated by qPCR on the prognostic significance of the mutation burden of *c-KIT*, an attempt to study the dynamic of the mutated clone has been done by Tan and colleagues [50]. They investigated the dynamic evolution of CBF-AML clones with *C-KIT* mutations by ddPCR combined with sequencing [50]. The study included 75 patients with CBF-AML, 19% of them with double *C-KIT* mutation.

Droplet digital PCR revealed that these double mutations can occur in either the same or different clones. Interestingly, according to the mutation, the clone was shown to present a different sensitivity to treatment. In particular, *C-KIT^N822^* mutation confers to the clone a better response to treatment as compared to *C-KIT^D816^* mutation. Moreover, D816 clone was the predominant clone at relapse. In addition, patients with double mutation had a better overall survival and event-free survival than those with a single mutation although the statistical significance was not reached, probably due to the small sample size. The study demonstrated that ddPCR is an effective method for monitoring clonal evolution in AML.

## 9. WT1 Assessment by Digital PCR as a Marker of MRD

The usefulness of *WT1* quantitative assessment q-PCR as a marker for measurable residual disease (MRD) detection in acute myeloid leukemia was demonstrated years ago [51,52,53,54,55]. Many studies clearly showed that *WT1* gene is overexpressed in about 80%–90% of patients affected by AML [51,52]. The persistence of *WT1* overexpression after chemotherapy is always indicative of persistence of leukemic cells [51,52,53,54,55]. Based on current evidence *WT1* can be considered a universal marker of AML. In a European study aimed at standardizing the method used for *WT1* measurement, it was shown that the clearance of the transcript to normal values is highly predictive of relapse [55]. Additional studies suggested that the persistence of abnormal *WT1* values after induction or consolidation impacts on the probability of relapse. An increase of *WT1* levels during follow up always predict leukemia recurrence [51,52,53,54,55]. The main advantages of *WT1* assay are that it can be measured in PB and that the method has been standardized [55].

Koizumi and colleagues [56] analyzed *WT1* by ddPCR and qPCR in 40 peripheral blood and bone marrow samples obtained from patients affected by acute leukemias and myelodysplastic syndromes. They found a strong correlation between the two methods (R = 0.99) but they demonstrated that dPCR is able to accurately detect lower *WT1* levels compared to qPCR. Based on these results the authors concluded that dPCR technology can be utilized to measure WT1 based MRD with high accuracy.

## 10. dPCR for MRD Detection in the Setting of Allogeneic Stem Cell Transplantation

Allogeneic hematopoietic stem cell transplantation is a consolidated therapy for the cure of patients with AML. Despite the significant number of patients achieving remission after chemotherapy and stem cell transplantation, the relapse rate is still significant. Many studies demonstrated that the presence of MRD both at the time of HSCT or after HSCT is a negative prognostic factor with high impact on survival [57,58]. Thus, nowadays, the detection of MRD to identify patients at high risk of relapse is mandatory. Different published studies addressed the issue of improving the sensitivity of PCR in order to increase the predictive value of the method. Bill and colleagues [59] analyzed by droplet dPCR the pre-transplant samples, both peripheral blood PB and bone marrow BM) of 51 nucleophosmin 1 (*NPM1*)-mutated AML patients transplanted in complete remission. The authors demonstrated that MRD positive patients have a higher cumulative incidence of relapse and shorter overall survival. In addition, the patients who are still positive for *NPM1* mutation by dPCR before allogeneic HSCT have a worse prognosis, independently from other known prognostic markers, or from the conditioning they received. The authors envisage the possibility of using dPCR routinely for patients with mutated *NPM1* to guide treatment and improve patients’ outcomes.

A larger study has been published by Valero-Garcia and colleagues [60]. They compared the sensitivity and accuracy of qPCR and dPCR and their capacity to predict relapse. They analyzed 28 adult patients who underwent allogeneic HSCT. They detected an increasing mixed chimerism prior to relapse in 100% of patients who relapsed. Compared with conventional qPCR, dPCR was able to predict relapse with a median anticipation period of 63 days versus 45.5 days by qPCR. Overall, 56% of the relapses were predicted earlier with dPCR whereas 38% of the relapses where detected simultaneously using both techniques. In only one case relapse was predicted earlier with qPCR. The presented data strongly support the notion that dPCR is a powerful tool to predict relapse after HSCT.

A summary of the main targets analyzed by dPCR and qPCR is shown in Table 1.

## 11. Chimerism Analysis

Finally, another important diagnostic tool in the setting of allogeneic HSCT is represented by chimerism analysis. For this purpose George and colleagues [61] set up a ddPCR assay and analyzed patients who received HSCT. With this method they detected the persistence of recipient cells with a high level of sensitivity. The techniques currently used to assess chimerism after hematopoietic stem cell transplantation are capillary electrophoresis analysis of STRs amplified by PCR and qPCR detection of polymorphic indel loci in the human genome. The main limit of capillary electrophoresis is the sensitivity. This method can detect chimerism fractions >1% of blood or marrow cells with the risk of missing clinically relevant low-level recipient cells. The second is a highly sensitive method with the disadvantage of being characterized by variable efficiency and the need for extensive validation and standards to be run with each assay [62,63,64].

The limit of capillary electrophoresis can be clinically relevant in the setting of cellular therapies that frequently result in microchimerism (donor chimerism <1%) [62].

George and colleagues described a highly sensitive droplet digital PCR assay with good performance throughout the range of clinically relevant chimerism measurements [64]. They validated the assay in serially diluted samples. The levels of detection and quantification of the assay were 0.01%.

## 12. Innovative Application of Digital PCR: The Case of Methylation Analysis

DNA methylation is an epigenetic modification that plays a key role in genome regulation [65]. Aberrant DNA methylation contributes to the genesis of tumors including hematological malignancies such as acute leukemia and myelodysplastic syndromes [66,67].

The regulation of CpG methylation has been demonstrated to play a role in stem cell maintenance and differentiation [68]. In hematological malignancies, global aberrant DNA methylation has been widely documented and it has been associated with disease progression and response to therapy [69,70]. Therefore, methylation analysis will help to understand the pathogenesis of leukemia and will represent new therapeutic targets. Alu sequences have been demonstrated to contribute to establish the epigenetic landscape of cancer cells, and several papers have been focused on this topic [71,72].

Orsini and colleagues [70] developed a new method of investigating Alu differential methylation, based on droplet digital PCR (ddPCR) technology. Although this method has not been applied to hematological malignancies and no data are available in this setting, this approach could be potentially useful to profile patients affected by hematologic disorders including myelodysplastic syndromes (MDS) for diagnostic and prognostic purposes.

## 13. Conclusions

A great deal of evidence has encouraged the investigation of dPCR for MRD monitoring in many hematological malignancies, especially in those patients who can reach very deep molecular responses with pharmacological or cellular therapies. Examples can be represented by the possibility to discontinue TKI therapy in CML patients or by the possibility of donor lymphocyte infusions in patients with detectable MRD after HSCT.

The real advantage of using dPCR will probably become clearer in the next few years with the improvement of the available strategies and the accumulation of data.

## Figures and Tables

**Table 1:** A summary of the main targets analyzed by dPCR and qPCR.

Disease	Target	Experiment	Monitoring
**CML**	Bcr-Abl	gene expression levels	MRD
**MPN**	Jak2^V617F^	DNA copies detection	MRD–HSCT
	CALR^mut^	DNA copies detection	MRD–HSCT
**APL**	PML/RARα	gene expression levels	MRD
**AML**	IDH1^mut^	DNA copies detection	MRD
	IDH2^mut^	DNA copies detection	MRD
	NPM1^mut^	DNA copies detection	MRD–HSCT

## Abbreviations

PCR	polymerase chain reaction
dNTP	deoxyribonucleotide triphosphates
dsDNA	double stranded DNA
ssDNA	single stranded DNA
qPCR	quantitative polymerase chain reaction
MFC	multiparameter flow cytometry
Ct	threshold cycle
dPCR	digital polymerase chain reaction
ddPCR	droplet digital digital polymerase chain reaction
CML	chronic myeloid leukemia
TKI	tyrosine kinase inhibitor
TFR	treatment free remission
MR	molecular remission
MR4	molecular remissionwith 4 logs of transcript reduction
MR4.5	Molecular remissionwith 4.5 logs of transcript reduction
ELN	European Leukemia Net
NCCN	National Comprehensive Cancer Network
rRQ-PCR	replicate quantitative polymerase chain reaction
MPN	myeloproliferative neoplasms
JAK2	janus kinase 2
CALR	calreticulin
ET	essential thrombocythemia
PMF	primary myelofibrosis
MRD	minimal residual disease
WT1	Wilms tumor gene
HSCT	hematopoietic stem cell transplantation
APL	acute promyelocytic leukemia
PML	promyelocytic leukemia
RARa	retinoic acid receptor alpha
ATRA	all trans retinoic acid
IDH1/2	Isocitrate dehydrogenase 1/2
NPM1	nucleophosmin
MDS	Myelodysplastic syndromes
